# The effect of cytomegalovirus infection on acute rejection in kidney transplanted patients

**DOI:** 10.15171/jrip.2016.18

**Published:** 2016-05-16

**Authors:** Boshra Hasanzamani, Maryam Hami, Vajihe Zolfaghari, Mahtab Torkamani, Mahin Ghorban Sabagh, Saiideh Ahmadi Simab

**Affiliations:** Kidney Transplantation Complications Research Center, Montaserie Organ Transplantation Hospital, School of Medicine, Mashhad University of Medical Sciences, Mashhad, Iran

**Keywords:** Cytomegalovirus, Acute rejection, Kidney transplantation

## Abstract

**Introduction:** It is known that cytomegalovirus (CMV) infection is a common problem among kidney transplant patients. This infection can be increased morbidity and decreased graft survival. This problem has been associated with acute rejection too.

**Patients and Methods:** One hundred and thirty renal transplant patients were included in a prospective, case-control study. The renal transplant patients were divided into two groups; patients group with CMV infection and control group without CMV infection. Serum CMV-IgG in all patients was positive (donor and recipients). None of patients had received anti-thymocyte-globulin and thymoglobulin. CMV infection was diagnosed by quantitative CMV-PCR (polymerase chain reaction) test (more than 500 copies/μg). Rejection episode was defined by kidney isotope scan or biopsy.

**Results:** In the group of 66 CMV infection patients (41 male [62.1%] and 25 female [37.9%]) the incidence of graft rejection was 36%, however in the group of 64 control patients the incidence of graft rejection was 9.4 % (*P* < 0.005).

**Conclusion:** CMV infection is important predisposing factor for acute allograft rejection after kidney transplantation. The results of this study suggests that the control of CMV infection could decrease episodes of acute kidney rejection.

**Implication for health policy/practice/research/medical education:**

Awareness about the relationship between cytomegalovirus (CMV) infection and acute renal rejection is very important. Early diagnose of CMV infection can reduce episodes of acute renal rejection.

## Introduction

It is known that cytomegalovirus (CMV) is a common pathogens that affects kidney transplant recipients (1).

If prophylaxis did not start, CMV infection usually occurs in the first month after transplantation. Exposure to the CMV can increase with age and present with positive CMV-IgG antibody. After transplantation, CMV infection can increase mortality and morbidity (2,3). Risk of CMV infection increased with administration of immunosuppressive drugs to prevent rejection. The CMV contamination may manifest as infection or disease. Detection of CMV by serology, culture or other techniques without sign and symptom of infection defined as CMV infection. If symptoms such as fever, leucopenia and fever or organ involvements occur, CMV disease is diagnosed.

## Objectives

The relationship between CMV infection and rejection is not well proven. Some studies have demonstrated the association of two disease, however, some other could not detect any relationship between two diseases (4). The cause of acute kidney transplant rejection after CMV infection is unknown, but immune modulation after infection can play as a major factor. There are some evidences suggest, CMV infection stimulates the immune system. Some studies indicate that CMV infection can induce up-regulation of adhesion molecules on endothelial cell of vessels. This up-regulation can enhance the inflammatory process (5).

In this study we investigated the relationship between CMV infection and acute rejection.

## Patients and Methods

Between November 2011 and May 2015, 130 renal allograft recipients were included in this case control study. The patients were divided into two groups: patients group, with CMV disease and control group, without CMV infection. Inclusion criteria were all transplanted patients older than 18 years old with CMV infection (CMV DNA levels more than 2000 copy per milliliters) in the patients group and without CMV infection in the control group. Exclusion criteria were patients had received polyclonal antibodies or experienced any episode of acute rejection. All donor and recipients were CMV seropositive prior transplantation. The kidney transplanted were from cadaver, unrelated or related donors. All patients received triple immunosuppressive therapy including calcineurin inhibitors, prednisolone and mycophenolate mofetil.

The CMV disease was suspected according to the present signs and symptoms such as; general manifestations (weakness, fever and leucopenia) and increase in hepatic transaminase or symptoms of respiratory involvement.

If CMV infection was suspected, blood sample was taken. CMV disease was diagnosed according to the quantitative CMV polymerase chain reaction more than 2000 copy per milliliters. For all patients with CMV viremia the antimetabolite stopped and treatment began with antiviral agents such as ganciclovir or valganciclovir and continued at least 21 days.

CMV induced acute rejection was considered if rejection happened within one month after infection. Allograft rejection suspected if any significant increase of baseline plasma creatinine, after ruling-out other causes of increased creatinine includes cyclosporine toxicity, pre-renal and post-renal acute kidney injury. Rejection was diagnosed by transplanted renal biopsy or renal scan. All biopsies reviewed by Banff classification (6). TC-99m diethylenetriaminepentaacetic acid (DTPA) scintigraphy is one of the method of diagnosing acute rejection. The results of which are comparable to biopsy (7). Delayed visualization of transplanted kidney, decreased perfusion, decreased and low parenchymal uptake is indicative of transplant rejection.

### Ethics issues

The study followed the tenets of the declaration of Helsinki. Informed consent was obtained from all patients. The research was approved by ethics committee of Mashhad University of Medical Sciences.

### Statistical analysis

Data were analyzed by SPSS software. Descriptive statistics including mean, frequency, SEM, percentage and analytic statistics including chi-square test, *t* test and Mann-Whitney U tests were utilized. *P* value less than 0.05 was considered as significant level.

## Results

In this case-control study, 130 kidney transplant patients were included.

Table 1, shows descriptive statistics, mean duration time of dialysis and cause of end-stage renal disease (ESRD) for the CMV infection group and control group. The mean age of patients in control and CMV groups was 36.16 and 36.91 years, respectively. 37.9% in control group and 39.1% in CMV group were female. Between two groups, the patients’ age, duration of dialysis, sex, cause of renal failure were not significantly different. In a group of 66 CMV disease patients the incidence of graft rejection was 36.4%, but in a group of 64 control patients, the incidence of graft rejection was 9.4%.

**Table 1:** Charactristics of kidney transplant recipients and prevelence of rejection in CMV infection and control group

**Variable**	**CMV infection**	**Control**	***P*** ** value**
Sex			0.89
Female	37.9%	39.1%	
Male	62.1%	60.9%	
Age			0.752
Mean ± SEM	36.91±1.67	36.16±16.77	
Duration of Dialysis			0.112
Mean ± SEM	33.59±2.72	32.09±1.32	
Rejection			0.000
No	63.6%	90.6%	
Yes	36.4%	9.4%	
Cause of ESRD			0.642
ADPKD	9.2%	7.8%	
GN	10.8%	7.8%	
HTN	26.2%	15.6%	
Diabetes	21.5%	25%	
Reflux	4.6%	10.9%	
Unknown	21.5%	26.6%	
Other	6.2%	6.2%	

Abbreviations: SEM, standard error of the mean; HTN, hypertension; ESRD, end-stage renal diseasel; CMV, cytomegalovirus; ADPKD, autosomal dominant polycystic kidney disease; GN , glomerulonephritis.

Table 2 shows the relationship between rejection and sex, age, duration of dialysis and cause of renal failure. In this study we found that, these parameters are not associated with transplant rejection.

**Table 2:** Correlation between main characteristics and CMV disease

**Variable**	**Rejection**		***P*** ** value**
	** CMV infection**	** Control**	
Sex			0.314
Female	33.3%	45.8%	
Male	66.7%	54.2%	
Age			0.366
Mean ± SEM	35.73±2	38.88±2.96	
Duration of dialysis			0.883
Mean ± SEM	33.29±3.56	34.13±4.22S	
Cause of ESRD			0.052
ADPKD	4.9%	16.7%	
GN	12.2%	8.3%	
HTN	24.4%	29.2%	
Diabetic	14.6%	33.3%	
Reflux	4.9%	4.2%	
Unknown	31.7%	4.2%	
Other	7.3%	4.2%	

Abbreviations: SEM, standard error of the mean; HTN, hypertension; ESRD, end-stage renal diseasel; CMV, cytomegalovirus.

## Discussion

CMV infection is one of the most common and important infection after kidney transplantation and important cause of mortality and morbidity. If prophylaxis against CMV is not started, CMV infection occurs early after kidney transplantation (commonly after first month) (8). Compared to other organ transplantation, kidney transplantation has the lowest risk for CMV infection (9). The most common risk factors for CMV infection include use of lymphocyte-depleting agents for induction or rejection therapy, donor-recipient mismatching and co-morbid infection and illness.

On the other hand, acute rejection is a major cause of allograft loss, and important predictor of chronic rejection. Acute allograft rejection is defined as an acute decrease in renal function. Commonly acute rejection occurs in the first 6 month after kidney transplantation (9).

In this study, we evaluated the association between CMV infection and acute renal allograft rejection.

We concluded that CMV disease is a risk factor for acute allograft rejection in patients with kidney transplantation.

Previous studies demonstrated that CMV disease is important risk factor for acute renal allograft rejection. Sagedal et al evaluated 477 kidney transplant patients and demonstrated that CMV disease is a predictor of rejection (10). Similarly, Toupance et al reported that CMV disease but not viremia, is a major risk factor for acute rejection in renal transplant recipients (11). However, this relationship has not been established in other studies. For example Michael et al, concluded that, after 5 years follow-up, CMV infection was not a risk factor for acute or chronic rejection (12).

CMV disease can cause dysregulation in immune system. This imbalance in the immune system may increase the risk of transplant rejection. Some studies on animal models found that CMV infection can augment the immune response and accelerated of collagen synthesis too (13).

## Conclusion

The results of our study showed that CMV disease can increase the risk of acute kidney transplant rejection, and factors controlling CMV infection, can reduce episode of acute rejection.

## Limitations of the study

This study had two limitations;

1-We had small sample size and suggest new study with large sample size.

2- Acute rejection in some patients but not all of them diagnosed with renal biopsy. We recommend to conduct kidney biopsy for all patients.

## Acknowledgments

We thank Mrs. Torkamani and Mrs. Zolfaghari, the staff nurse of the Montaseriye Transplant Center, who cooperate to this study.

## Authors’ contribution

BH; design, data collection, literature search, manuscript writing. MH and MG; helped with patient management and deci­sions towards management. VZ and MT; collected the data. SA; performed the data analysis. BH; edited the final manuscript. All the authors reviewed and approved the manuscript.

## Conflicts of interest

The authors declared no competing interests.

## Ethical considerations

Plagiarism, fabrication and other ethical issues have been observed by the authors.

## Funding/Support

This study with approval No.922412 issued by Mashhad University of Medical Science and Kidney Transplantation Complications Research Center.
